# The Effectiveness of Probiotics in the Treatment of Inflammatory Bowel Disease (IBD)—A Critical Review

**DOI:** 10.3390/nu12071973

**Published:** 2020-07-02

**Authors:** Dominika Jakubczyk, Katarzyna Leszczyńska, Sabina Górska

**Affiliations:** Laboratory of Microbiome Immunobiology, Institute of Immunology and Experimental Therapy, Polish Academy of Sciences, Weigla 12, 53-114 Wroclaw, Dolnoslaskie, Poland; katarzyna.leszczynska@hirszfeld.pl (K.L.); sabina.gorska@hirszfeld.pl (S.G.)

**Keywords:** probiotic, inflammatory bowel disease, *Bifidobacterium*, Crohn’s disease, ulcerative colitis, treatment, anti-inflammatory

## Abstract

Inflammatory bowel disease (IBD), which affects millions of people worldwide, includes two separate diseases: Crohn’s disease (CD) and ulcerative colitis (UC). Although the background (chronic inflammatory state) and some of the symptoms of CD and UC are similar, both diseases differ from each other. It is becoming clear that a combination of many factors, in particular genetic background, host immune response and microbial reduced diversity status are associated with IBD. One potential strategy to prevent/treat IBD is gut modulation by probiotics. Over the last twenty years, many publications have focused on the role of probiotics in the course of IBD. The review discusses the utility of different strains of probiotics, especially *Bifidobacterium* spp., in all factors potentially involved in the etiology of IBD. The probiotic modulatory properties among different study models (cell lines, animal models of colitis, clinical study) are discussed and probiotic usefulness is assessed in relation to the treatment, prevention, and remission of diseases.

## 1. Introduction

Diseases within the gastrointestinal (GI) tract were known even in ancient times, and most likely, Hippocrates was the first to try to diagnose the causes of diarrhea [1]. The term IBD (inflammatory bowel disease) was coined in the 1970s, specifically to unify the understanding of diseases within the GI tract. IBD, as a common appellation, describes chronic inflammatory states in the gastrointestinal tract. This term includes two different clinical states: Crohn’s disease (CD) and ulcerative colitis (UC). The first description of UC dates from 1859, when Samuel Wilks used the term ‘ulcerative colitis’ [2,3]. CD was named after Burrill B. Crohn, who in 1932, together with Leon Ginzburg and Gordon D. Oppenheimer, introduced a description of the disease [3]. Initially, IBD was associated with highly developed countries. However, currently, the increase in new cases has been observed worldwide. Generally, it is estimated that three million people in Europe [4], three million Americans [5], and more than 80,000 Australians [6] are affected by IBD. Additionally, increasingly, new cases are observed in Asia and Africa [7,8]. The exact causes of IBD are still unknown. The disease is characterized by states of exacerbation and remission, and one of the therapeutic goals is to maintain the possibly long remission phase. Over the last twenty years, many publications have focused on the role of probiotics in the course of IBD. The aim of this review is a general survey of the literature over the last two decades concerning probiotics and their influence on IBD. The review discusses the utility of different strains of probiotics, especially *Bifidobacterium* spp, in all factors potentially involved in the etiology of IBD. The search of the review literature was based on the muster of publications from the last twenty years. The publications included had to concern IBD and probiotics (bacterial cells, bacterial components, and/or bacterial metabolites) and be research articles.

## 2. Clinical Picture of Inflammatory Bowel Disease

Although the background (chronic inflammatory state) and some of the symptoms of CD and UC are similar, both diseases differ from each other. Exact diagnosis is necessary for correct treatment. Crohn’s disease can affect all parts of the digestive tract, from the oral cavity to the anus. It has a discontinuous character (inflamed tissue occurs alternately with non-inflamed parts). The changes are spread within the whole structure of the bowel wall. Characteristics also include granuloma changes. The consensus referring to the phenotype of CD among adult patients was established in Montreal (2005) [9] and it divides the disease according to age, location, and behavior over time. Based on Montreal’s classification, the pediatric grouping criteria were established in 2011 in Paris including changes in growth [10]. In contrast to Crohn’s disease, ulcerative colitis has a continuous character. It is limited to the colon area, and inflammation changes are spread within the inner part of the mucosa. The Montreal classification for UC includes the criteria of severity and extensity. Both classifications are shown in Table 1.

The occurrence of UC and CD can be compared to a sine wave, periods of symptom intensification interweave with a remission phase. Commonly shared symptoms for CD and UC are diarrhea (also with bleeding), obstruction, abdominal cramps and/or pain, loss of weight, fever, weakness, fatigue, and malnutrition. In the course of IBD, extra manifestations can also be observed and more interestingly, uncharacteristic symptoms contribute to an earlier identification of the disease in about 25% patients with early changes within the GI tract [12]. Extraintestinal manifestations of IBD include among others pulmonary symptoms (unknown prevalence) [13], arthropathy (prevalence 17–39%) [14], and eye diseases (up to 72% of IBD patients) [15]. Nevertheless, the frequency of additional symptoms differs between UD and CD groups. The cohort study made by Isene and co-workers enrolled 1145 patients with IBD from Europe and Israel, and continued observations for 10 years. As a result, the authors noted that patients with Crohn disease showed twice the exposure to arthropathy, eye, skin, and liver changes as patients with UC [16]. Cutaneous extraintestinal manifestations are usually divided by their pathogenesis and consist of five groups: granulomatous, reactive form, immunologically associated, the outcome of nutritional deficiencies, and the outcome of treatment [17]. Vide and co-workers performed a cohort-study within 342 patients with CD (62%) and UC (38%) and estimated that cutaneous extraintestinal manifestation such as granulomas concerned 0.3% of patients, a reactive form was noted in up to 4.4%, an immunologically associated form in 10.5%, nutritional deficiencies in up to 6.4%, and changes as an outcome of therapy in 29.5% [18]. Additionally, the cutaneous symptoms more often manifested among patients with CD, and regarding the UC patients, those with the E3 form of the disease [18].

## 3. The Key-Players in Inflammatory Bowel Disease (IBD)

**Figure 1:**
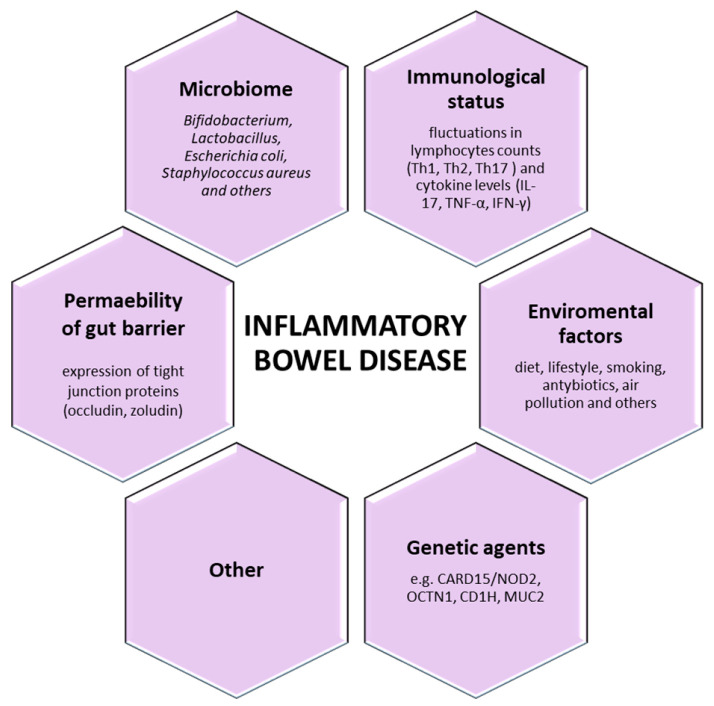
The key players in inflammatory bowel disease.

Coexistence of environmental and genetic agents, immunological imbalance, permeability of gut barrier, and state of the microbiome have a great impact on the rise and progress of the disease. However, the direct mechanism and cause of IBD remain unclear. The key players in IBD are shown in Figure 1.

### 3.1. Genetic Aspect

Genetic background in IBD is an important factor in the course of the disease. In 2012, Jostins et al. published genome-wide association studies and meta-analyses of 75,000 individuals with CD, UC, and control groups, and found 163 IBD loci (around 300 genes) directly associated with the disease [19]. Interestingly, 30 loci were characteristic only to CD, 23 loci only for UC, and 110 loci were associated with both IBD subtypes [19]. Three years later, Liu et al. published another genetic study taking into account population ancestry. Researchers included 86,682 European participants and 9846 individuals from Iran, India, and East Asia. Thirty-eight new loci were found to be connected with IBD, which increased the total number of loci associated with IBD occurrence to 200. Some of the loci were characteristic to the ancestry of the population [20]. Additionally, both studies underline that genetic factors, only together with other stimuli, can lead to the development of the disease.

### 3.2. Environmental Aspect

In the literature, there are contrary data about the meaning of the environmental factors. Among others, one of the most important environmental agents seems to be diet and lifestyle. It was noted that long term intake of trans-unsaturated fats, low intake of fruits, vegetables, and fish are associated with a high risk of IBD, but in different grades relating to UC or CD [21,22,23,24]. A diet rich in sugar, soft drinks, and a low intake of vegetables is more correlated with UC [24], whereas high red meat and cheese consumption is more associated with CD [25]. The association of alcohol abuse and the occurrence of UC or CD was not observed [26]. Smoking seems to be an aggravating factor for CD, but protective for patients with UC [22,27]. Some of the study points to the possibility of a correlation between the use of some drugs (NSAIDs, oral contraceptive pills) and an escalation of the IBD symptoms [28,29,30]. Air pollution seems to be important in increasing the early onset of both diseases and could have an impact on the numbers hospitalized among patients with IBD [31,32]. Due to changes within the GI tract’s microbiota, bacterial infection was suspected of contributing to IBD development. Nonetheless, only a few reports can be found that confirm direct bacterial involvement in IBD pathoetiology. Tripathi and co-workers found a link between a *Salmonella* infection and UC. Using the nested PCR method, they identified that 80% of patients with UC had a positive result for the *Salmonella enterica* serotypes (Typhi and Paratyphi [33]). Moreover, the presence of *Clostridium difficile* among UC and CD patients was correlated with longer hospitalization, worse prognosis, and a higher rate of death [34,35,36]. Nevertheless, the authors of all articles conclude that further research is needed to elucidate the bacterial influence on IBD. Interestingly, helminthic infections could have some influence on IBD, which seems to follow the ‘IBD hygiene theory’. Parasites change the microbiota landscape and modulate the immunologic status of the organism [37,38,39]. This contributes to the conclusion that helminths can form a new therapeutic strategy [40,41]. Further research is needed to estimate if helminth-based therapy can be included in the IBD treatment strategy.

### 3.3. Immunological Aspects

**Figure 2:**
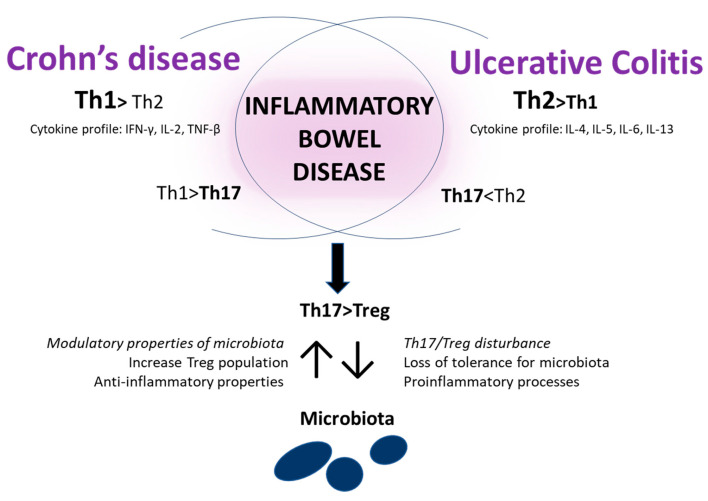
The role of the immune response in IBD. In CD, Th1 skewing is observed, but in UD, Th2 skewing is common. The population of Th17 cells is decreased regarding the Th1 or Th2 population. The decreased number of Treg cells and imbalance in Th17 and Treg subpopulation are common in both diseases. The subpopulation disturbance triggers the loss of tolerance for microbiota and the privilege of the proinflammatory processes. In turn, microbiota modulates the Treg population and has an anti-inflammatory effect.

Another key player in IBD is the immunological status of the organism. The most important cells seem to be helper lymphocytes Th (Th1, Th2, Th17) and regulatory T cells. It has been described that CD is connected with Th1 skewing, while UC with Th2 [42,43,44,45]. Generally, the polarization toward Th1 or Th2 depends on the cytokine profile produced as an effect of microorganism recognition by MAMPs (microbe-associated molecular patterns). Th1 cells produce IFN-gamma, IL-2, and TNF-beta, which are mainly associated with intracellular bacteria and virus killing. Th2 cells produce IL-4, IL-5, IL-6, and IL-13, which are usually linked with parasitic worms and allergic reaction. For both diseases, activity of the Th17 cells seems to be crucial and reside on the gut’s mucosa. Th17 cells produce IL-17, TNF-alpha, IL-22, and INF-gamma. IL-17 is a predominant cytokine involved in extracellular pathogen defense. It is not able to block the Th1 and Th2 cells, which explains the skewing Th1/Th17 or Th2/Th17 observed in IBD [46]. Generally, IL-17 is recognized as pro-inflammatory. Nevertheless, it has subtypes that have a different meaning for the host. A few studies have demonstrated that the blocking of Th17 derived IL-17A increased the dextran sodium sulfate (DSS)-colitis symptoms (a protective meaning), and IL-17F deficiency relieved the symptoms of colitis (a proinflammatory meaning) [47,48]. Similar to the Th17 cells, the peripherally induced Treg cells (iTreg), which have a similar formation pathway like Th17, also reside within the gut mucosa, where microbiota tolerance induction takes place. The main role of Treg is controlling the Th cells and protecting against their products’ overexpression. Interestingly, iTreg cells, after appropriate stimulation (IL-6), can not only induce a rise in Th17 population, but can also differentiate themselves from Th17 [49]. The peripherally induced Treg can arise in contact with digested nutritional substances such as retinoic acid or intestinal microbiota [50,51]. In the course of IBD, the gut microbiota is disturbed. It was also noted that the balance between the Th17 and Treg is changed (Th17/Treg), and the numbers of Treg among IBD patients decreases [52]. This leads to the conclusion that a downregulation of Treg and an increase of Th17 cells are a cause of the loss of tolerance for microbiota and the initiation of proinflammatory processes. However, it should be noted that microbiota is not only a passive player, and it can also modulate the T cells. Mazmanian and co-workers proved that in a physiological state, the commensal bacteria regulate the functions of Treg and Th17 cells. The polysaccharide A derived from *Bacteroides fragilis* decreased the amount of pro-inflammatory IL-17 and protected from the colitis induced by *Helicobacter hepaticus* in an animal experimental model [53]. Atarashi and co-workers published a report describing that the induction of Treg cells is conditional on *Clostridium* metabolites, and their identification allows one to estimate a future therapeutic option [51]. Additionally, the gut microbiota produces host-beneficial substances. Among IBD patients, changes in gut microbiota composition resulted in a decreased level of short-chain fatty acids (SCFA), which have anti-inflammatory properties and are one of the energy sources for colon cells [54]. The role of the immune response in IBD is shown in Figure 2.

### 3.4. The Gut Barrier and Microbiota

Another important aspect of IBD is the gut’s barrier between the internal and external environment. The gut is exposed to many factors (food antigens, residence microbiota, pathogenic organisms, etc.). The role of this barrier is double-sided. First, it prevents the penetration of unwanted antigens; second, it passes some of the nutrient substances through the intestinal wall. In IBD, this barrier is disturbed and its permeability is increased. The direct causes of augmented permeability are unknown. In the literature, one can find reports describing a genetic association with this phenomenon. The changes within *CARD15/NOD2, OCTN1, CD1H*, and *C1ORF106*, connected with tight junctions (TJ), cationic channels, recognition of microorganisms, etc., translate to a decrease in the robustness of the gut barrier [55,56,57,58,59]. Nevertheless, a few studies have checked the connection between genetic factors and gut permeability among healthy first-degree relatives of CD patients. Surprisingly, the dependency was not proven, which can indicate other strong factors involved in increasing gut permeability [60,61]. Pro-inflammatory cytokines such as IL-13, TNF family members, and IFN-γ [62] can modulate the expression of the tight junctions [63,64]. Microbiome products and/or antigens not only affect the immune cells, but can also have a direct impact on the connection of the epithelial cells. Carlsson and co-workers proved that products from *Faecalibacterium prausnitzii* improved the tightness of the gut barrier among mice with DSS-induced colitis [65]. Laval and co-workers published data that compared the effect of *Faecalibacterium prausnitzii* with probiotic *Lactobacillus rhamnosus* CNCM I-3690 on the cell line modes and the mice model. They concluded that both strains had similar properties, and *L. rhamnosus* increased the occludin and E-cadherin proteins [66].

## 4. Probiotics. Treatment and Protection from IBD

Probiotic bacteria have a positive impact on the host organism when administrated in an appropriate proportion. The usefulness of probiotics is known through antibiotic-based therapy [67,68] to decrease blood cholesterol level [69], the treatment of local infections [70], and others. Probiotics have properties for the immunomodulation of many processes [71]. Nevertheless, the exact path of the influence, mechanisms, and structures involved are still unknown. In the literature, there are reports of the usefulness of probiotics in reference to IBD. However, there is still a lack of coherent opinion concerning their exact utility. The idea of treating and preventing IBD with a probiotic is catching on. There have been many reports providing evidence that some probiotic strains can be useful during treatment and prevention against IBD, both in the murine and rat models of the disease. The results, even though approximate, indicate some differences, probably triggered by the research methods selected and/or the selected bacterial strain. In the animal model for colitis induction, DSS (dextran sulfate sodium) or TNBS (2,4,6-trinitrobenzene sulfonic acid) are the most common. The usage of DSS is dictated by its strong, rapid, and dose-dependent UC-like effect [72]. TNBS is widely used to trigger a CD-like colitis [73]. These models do not fully reflect human IBD; nevertheless, up to now, they are the best known colitis stimuli [74].

### 4.1. Probiotic Effectiveness in Animal Model of Colitis

Javed and co-workers [75] showed the beneficial effect of *Bifidobacterium infantis* on the reduction of colitis induced by TNBS (2,4,6-trinitrobenzene sulfonic acid). Among the group of rats with colitis and supplemented with *Bifidobacterium infantis*, researchers noted a reduction in symptoms, weaker damage to the mucosal architecture, which indicated the protective meaning of probiotics for mucus goblet cells and the epithelial cell layer. In the murine model of TNBS colitis, oral supplementation with *Bifidobacterium bifidum* reduced the course of the disease on issues of colonic edema, macroscopic damage, histological scores, and additionally seemed to prevent weight-loss [76,77]. Based on another research group, supplementation with *Bifidobacterium bifidum* significantly increased the level of IL-10 and reduced the level of IL-1β in the colon sections, which confirmed the anti-inflammatory effect [77]. Those findings seem to confirm that *Bifidobacterium infantis* and *Bifidobacterium bifidum* have modulatory properties, and reduce the inflammation as well as the clinical symptoms of colitis. Nevertheless, it seems clear that not all strains of probiotics have an effect. In the hapten model of colitis (TNBS) run by Kenned and co-workers, no beneficial effect of *Lactobacillus plantarum* species 299 was observed on the rat’s gut permeability, weight changes, colon microscopic scores, and the level of blood albumins [78], which are contrary to some of the other available reports. This can be caused by a dose of TNBS (30 mg), the severity of the induced colitis (which could be irreversible at some point) as well as the properties of the single bacterial strain, which modulate their environment in many different modes. Instead, in the murine model of DSS-colitis, the beneficial effects of *Bifidobacterium* strains were reported. It was proven that *Bifidobacterium animalis* subsp. *lactis* BB12 and *Bifidobacterium longum* subsp. *infantis* BB-02 alleviated both susceptibility to and symptoms of the disease [79,80]. A group of mice supplemented with *Bifidobacterium animalis* subsp. *lactis* BB12 was protected from a reduction in colon length and had a better picture of the colon’s histology. Moreover, the reduction in apoptosis in the IECs (intestinal epithelial cells) and a decrease in the level of TNF-α was observed [79]. *Bifidobacterium longum* subsp. *infantis* BB-02 attenuated the clinical symptoms of the disease, protected the colonic structure and reduced edema compared to the non-probiotic supplemented group [80]. The study conducted on the T-bet-/-Rag2-/- ulcerative colitis mouse model indicated that *Bifidobacterium lactis* reduced colitis and inflammation in an early stage of the disease and decreased the level of *Enterobacteriaceae*, which seems to be colitogenic [81]. Another published report indicates that dairy-derived *Lactobacillus delbrueckii* modulated the Nuclear Factor kappa-light-chain-enhancer of activated B cells (NF-kB) pathway and reduced the inflammatory state in the DSS-colitis mice model [82]. This implies that probiotic strains can differ in their immunomodulation properties. These differences can be the result of the experimental conditions such as the animal model (rats, mice), the chosen inductor of colitis (DSS or TNBS), the severity of colitis (dose-dependent) as well as differences between the bacterial strains. It is worth mentioning that environmental conditions and the microbiota composition also modulate the bacteria properties. Traina and co-workers [83] presented a report that estimated the influence of TNBS on the gut microflora. In the murine model, three days after the injection of 150 mg/mouse TNBS in 50% ethanol, the authors noted an increase in E. coli and *Clostridium* spp. populations and a decrease in *Bifidobacterium* and *Lactobacillus* strains. The altered bacterial microflora could be one of the reasons why some of the studies failed. There are many reports confirming that the positive effects of probiotics can be obtained only in cases of consuming a mixture of different strains. However, probiotic as mixtures of different strains will be discussed further. Another point is that probiotic properties can be strongly dependent on the metabolic activity of the strains. Biagoli and co-workers [84] ran a comparison study that checked the beneficial effects of the probiotic mixtures (VSL#3) in the murine models DSS and TNBS induced colitis. Mixtures of probiotics containing the same strains (according to the label, the first mixture contained *Streptococcus thermophilus* DSM 24731, *Bifidobacterium longum* DSM 24736, *Bifidobacterium breve* DSM 24732, *Bifidobacterium infantis* DSM 24737, *Lactobacillus acidophilus* DSM 24735, *Lactobacillus plantarum* DSM 24730, *Lactobacillus paracasei* DSM 24733, and *Lactobacillus debrueckii* subsp. *bulgaricus* DSM 24734; the second mixture contained *Streptococcus thermophilus* BT01, *Bifidobacterium. breve* BB02, *Bifidobacterium longum* BL03, *Bifidobacterium infantis* BI04, *Lactobacillus acidophilus* BA05, *Lactobacillus plantarum* BP06, *Lactobacillus paracasei* BP07, and *Lactobacillus debrueckii* subsp. *bulgaricus* BD08) were produced by two different manufacturers and available under the same brand. Surprisingly, only one of the mixtures had a beneficial effect on colitis in both models. The second mixture redounded to a worsening of the inflammatory state, as expressed by clinical scores, length, and weight of the colon and the permeability of the tight junctions. In the report, the difference between colitis models was also visible, nevertheless, it was not a subject of assessment. Another research group headed by Hrdy [85] proved that probiotic strains affected host cells in many ways. The study was based on the murine model of TNBS-induced colitis, and the mechanism of action of *Bifidobacterium animalis* spp. *lactis* Bl 5764 and *Lactobacillus reuteri* Lr 5454 was determined. It was indicated that both of the strains had a beneficial effect on the host, which was expressed through body weight, macroscopic indicators of inflammation (Wallace scores), and histopathological analysis (Ameho score) as well as the level of lipocalin-2 in the feces. Moreover, a different influence was observed on the dendritic cells (DC). Strain Lr 5454 was more involved in the development of tolerogenic DC and induced Tregs population and expression of Reg3b in a NOD2-independent manner. In contrast, Bl 5764 promoted bone marrow-derived dendritic cell maturation and IL-17A secretion. All of the above studies led to the conclusion that even in a simplified animal model of colitis, which skips the genetic and external environmental influence, a broad and multidisciplinary approach is needed.

### 4.2. Clinical Study of Probiotic among IBD Patients

Within the patient-based studies, more differentiated results are noticeable. Among CD patients, administration of *Saccharomyces boulardii* was helpful in maintaining remission and bowel sealing [86]. With regard to UC, the strains *Escherichia coli* Nissle1917, *Bifidobacterium breve*, *Bifidobacterium bifidum*, and *Lactobacillus acidophilus* seem to be promising in sustaining the remission phase [87,88]. Administration of *Lactobacillus* fermentum among UC patients resulted in NF-kB lowering regulation and additionally decreased the IL-6 and TNF-alpha levels [89]. Groeger and co-workers [90] showed that oral administration of *Bifidobacterium infantis* 35,624 reduced the levels of C-reactive protein (CRP) and TNF-α in both gastrointestinal and non-gastrointestinal inflammatory disorders, but did not particularly affect UC disease. Similar results were shown by Ishikawa and co-workers [91]. Patient groups with UC and supplemented with *Bifidobacterium breve* strain *Yakult* elicited better endoscopic scores in comparison to the group without supplementation. Nevertheless, Matsuoka and co-workers [92] did not confirm these results. They demonstrated the lack of a *Bifidobacterium breve* strain *Yakult* effect on the maintenance of remission in UC patients. The authors did not observe important differences even among patients treated for 48 weeks with a mixture of *Bifidobacterium breve* strain *Yakult* and *Lactobacillus acidophilus*. The conclusions were made by the authors based on clinical symptoms and stool sample determination as they did not perform the endoscopic analysis [92]. Discrepant results upon supplementation with the same probiotic strains can be triggered by the activity of the bacteria as well as through the influences of other strains present in the host organism. This can explain why the use of a mixture of the respective strains indicates better effects than using one strain alone.

The probiotic mixture of *Lactobacillus acidophilus* strain LA-5 and *Bifidobacterium animalis* subsp. *lactis* BB12 (Probio-Tec AB25) was examined among patients with UC disease (32 patients). The results indicated a maintained remission in up to 25% (five patents) of treated patients and up to 8% (one patient) in the placebo group. Those changes were not statistically significant (*p* > 0.37). Days to relapse were also insignificant (respectively 124 vs. 104, *p* > 0.68). This indicated that Probio-Tec AB25 was not effective enough for UC patients [93]. However, Tamaki and co-workers [94] showed that the treatment of patients with mild to moderate UC using *Bifidobacterium longum* 536 significantly decreased not only the disease activity index and downscaled the rectal bleeding, but those patients also achieved a clinical remission. Treatment with probiotics together with commonly used anti-inflammatory drugs seems to be a more effective solution in comparison to treatment with probiotics alone. Palumbo and co-workers [95] demonstrated the positive effect of mesalazine use (Mesavancol^®^ 1200 mg CPR, Giuliani spa, Milan, Italy) with the probiotic mix (*Lactobacillus salivarius, Lactobacillus acidophilus,* and *Bifidobacterium bifidum* strain BGN4; Acronelle^®^, Bromatech SRL, Milan, Italy) among UC patients. The group with the double treatment showed reduced recovery time, weaker activity of the disease, and they presented a better endoscopic picture. The most common probiotic cocktail of proven efficacy is VSL#3. This mixture contains 900 billion lyophilized bacteria, comprising four strains of *Lactobacillus* (*L. paracasei, L. plantarum, L. acidophilus*, and *L. delbrueckii* subspecies *bulgaricus*), three strains of *Bifidobacteria* (*B. longum, B. breve*, and *B. infantis*), and one strain of *Streptococcus thermophilus* [96,97,98,99]. The efficacy of VSL#3 was proven in the DSS-induced colitis mice model [96] and in patients with mild to moderate active UC colitis [97]. Wang and co-workers showed that treatment of mice with the DSS-induced colitis by 5-ASA, VSL#3, or both 5-ASA and VSL#3 decreased the level of TNF-α and IL-6. Bacterial supplementation reduced the number of pathogenic microbiota and increased the population of *Bifidobacterium* and other non-pathogenic species in the intestinal mucosa [96]. Sood and co-workers ran a randomized, double-blind placebo-controlled trial that proved the beneficial effect of VSL#3 [97]. A group of adult patients with a mild to moderately active form of UC was supplemented with VSL#3 twice daily for 12 weeks. Results of the treatment were measured by the UCDAI score. The authors noted a remission caused by probiotic use. Patients in the VSL#3 group had a significant improvement in rectal bleeding and stool frequency, mucosal appearance, and overall physician’s evaluation [97]. It must be noted that more than 92% of patients received additional anti-inflammatory medication (immunosuppressants and/or mesalamine) as a part of the routine therapy, which can have an influence on the results obtained. The observed improvement can arise not only from the probiotic side, but can be a result of synergy between standard drugs and VSL#3. In another study, Tursi and co-workers checked the VLS#3 as an adjunct to the routinely used pharmaceutical treatment [98]. After eight weeks of supplementation with the probiotic mixture, a reduction in UCDAI scores and frequency of rectal bleeding was observed, but there were no statistical differences in parameters such as the physician’s rate of disease activity or endoscopic scores. These results suggest that a beneficial effect of probiotics eventuates from long-term use.

The effectiveness of VLS#3 was confirmed also in reference to the child population (mean age 12) with mild-to-moderate UC. Huynh and co-workers [100] evaluated the simple clinical colitis activity index (SCCAI), Mayo ulcerative colitis endoscopic score, and some of the inflammatory markers (ESR, CRP, interleukin level) and rectal tissue microbial profiling, which was done at baseline, and at week 8 of supplementation with VSL#3. The authors reported that 56% of patients indicated a remission, 67% of patients in remission improved their microbiota composition, and IFN-gamma, TNF-alpha, CRP, and ESR decreased.

However, the VSL#3 supply of patients with CD was not as effective as for UC. Still, the sooner the therapy started, the better the results obtained [101]. Although intestinal microflora changes in the case of CD including a decrease in the population of *Bifidobacterium* strains [102], supplementation with a probiotic cocktail had only a small effect on inhibiting endoscopic recurrence [101]. Supplementation with other strains such as *Faecalibacterium prausnitzii* seems to be more useful in the case of Crohn’s disease, which has been confirmed by Sokol and co-workers [103].

It should be noted that different strains of the bacteria can have a different function in and relation to the host. The previous study showed that probiotics can affect different aspects of IBD. It must be mentioned that IBD is a multi-factor disease, so to distinguish one beneficial strain for patients with the same disease seems to be impossible. The anti-inflammatory effect is highly dose- and strain-dependent. Further research is needed to estimate the appropriate probiotic-based therapy, but it seems to be clear that it is impossible to determine one universal product beneficial for all inflammatory-based diseases. Clearly, personalized medicine is needed here. The type of inflammatory changes, the severity of the disease, the microbiota composition as well as environmental and genetic aspects should be considered. For these reasons, reports about the usefulness of the living strains among patients can vary. Until now, live probiotic bacteria have been perceived to be the most beneficial to the host.

### 4.3. Probiotic Bacteria and IBD-Associated Cancer

IBD is based on a chronic inflammatory state which is one of the risk factors for malignancy development. In clinical practice, colorectal cancer, small bowel cancer, primary intestinal lymphoproliferative disorders, cholangiocarcinoma, and other neoplastic processes can occur [104,105,106,107]. The usefulness of the probiotic strains as prevention from neoplasia among IBD patients is a subject of analyses. Seung and co-workers [108], based on the cell line and murine model, indicated that the presence of *Bifidobacterium lactis* in a pro-inflammatory stimulated cell line decreased activity of the NF-κB. In the murine model of cancer, the presence of *Bifidobacterium lactis* was related to the decreased activity of the NF-κB and the improvement of the clinical picture (with lower number and size of tumors in comparison to the mice group with cancer, but not supplemented with *Bifidobacterium*). In another murine based model, the VSL#3 immunomodulatory properties referring to colorectal cancer were tested. Riera and coworkers [109] supplemented the mice with VSL#3 (1.2 billion bacteria per mouse/day) or conjugated linoleic acid (CLA). Cancer was induced by azoxymethane, DSS (the first step), and a single dose of 5 × 10^7^ CFU *Helicobacter typhlonius* by oral gavage (the second step cancer induction). Observation took 68 days. It was indicated that animals treated by CLA or VSL#3 had a shorter recovery time, and lower disease severity in an active phase of cancer. VSL#3 treatment was related to a higher mRNA expression of TNF-α and increase of the angiostatin level, IL-17 expression among CD4+T cells in mesenteric lymph nodes, Treg in lamina proportia as well as memory T cells. The treatment of CLA decreased the level of COX-2. In another study, the beneficial effects of *Lactobacillus acidophilus* and *Lactobacillus fermentum* were also indicated. In a murine model of colon cancer, it was indicated that these two strains, especially in connection, presented antioxidant, antiproliferative, and pro-apoptotic activities for cancer [110]. These features seem to be very promising, especially for the reduction of the uncontrolled growing process of cancer cells. According to the available data, probiotic pretreatment can delay or even withhold the carcinoma processes. Appleyard and co-workers supplemented the rats with VSL#3 one week before the colorectal cancer induction by TNBS. Then, the rats were supplemented with probiotics in drinking water until the end of the experiment (17 weeks) [111]. VSL#3 improved the histological picture of the colon, and increased the level of angiostatin and vitamin D receptor (VDR), which also have an anti-tumor effect. Nevertheless, Janelle and co-workers did not confirm the beneficial effect of probiotic supplementation in a cancer model [112]. The authors used a well-described VSL#3 probiotic mixture for supplementation of IL-10-deficient mice with colitis-associated cancer induced by azoxymethane. The authors noted that VSL#3 did not secure against inflammatory processes and tumor development increased the tumor penetrance and made histologic dysplasia scores worse. Moreover, supplementation with VSL#3 extended the *Clostridium* population. Among the human study, it was indicated that the probiotic bacteria can be beneficial, especially after colorectal surgery. A randomized, double-blind, a placebo-controlled study indicated that supplementation with *Lactobacillus acidophilus*, *L. plantarum, Bifidobacterium lactis*, and *Saccharomyces boulardii* one day before the surgery and continuing for another 15 days postoperatively, reduced the rate of postoperative pneumonia, surgical site infections, and anastomotic leakage [113]. In the trial study run by Hibberd and co-workers, patients with colorectal cancer were treated with a mixture of 1.4 × 10^10^ CFUs *Bifidobacterium lactis* Bl-04 and 7 × 10^9^ CFUs *Lactobacillus acidophilus* for 8–78 days [114]. Results showed that the usage of probiotics changed the microbiota composition and genera associated with cancer (*Fusobacterium, Peptostreptococcus*) were reduced.

The discrepant results can arise from the research protocol scheme. The experiments conducted indicate that in the animal model of diseases (colitis and cancer as well), the probiotic pretreatment, before the pathological state was induced, gave better results. This suggests that the dispersion of gut microflora is one of the basic protective elements. Another thing is the choice of the animal model. Animal studies do not fully reflect human immunology (especially in a multi-factored diseases) and the genus of mouse or rat can also be different from each other [115]. This indicates that the usefulness of the probiotic should be tested on many different models.

### 4.4. The Effect of Components and Metabolites Produce by Probiotic Strains

Nevertheless, the advanced research indicates that the influence of bacteria on the eukaryotic cells is more complex. Simultaneously, there is a growing body of evidence that separate structures of bacteria have a great impact on the host. There are more and more reports that the interaction between bacteria and host takes place via the respective cell components. Deutsch and co-workers [116] determined the proteins with anti-inflammatory properties deriving from probiotic cheese strain, *Propionibacterium freudenreichii*. They distinguished twelve strains for proteomic study, and then particularized eight for further transcriptomic analyses. Through the gen-inactivation validation method, researchers determined a few surface proteins (SlpB, SlpE, two proteins with SLH domains, HsdM3,) potentially connected with an IL-10 increase and anti-inflammatory value. Moreover, according to the authors, the anti-inflammatory properties were kept only for the specific configuration of the components, never for single proteins, and are highly strain dependent. S.R. Qi and co-workers [117] published a report that indicated the importance of the individual components to the cell line treated with LPS. The macrophage cell line RAW 264.7 with the induced inflammatory state was treated with a surface layer protein, genomic DNA, and unmethylated cytosine–phosphate–guanine-containing oligodeoxynucleotides, alone or in combination. These components were isolated from *Lactobacillus rhamnosus* GG. The authors estimated the immunomodulation properties of each component through an assessment of the expression of the toll-like receptors-2, -4, and -9, mitogen-activated protein kinases, and nuclear factor-kappa B signaling pathways. The researchers indicated that in an inflammatory condition, the above components suppress the respective inflammatory paths which present in IL-6 and TNF-α decreasing. However, they did not observe particular differences between single and mixed components.

Not only the full strains and cell components, but also the bacteria’s cell-free growth medium contains components that can stimulate the host cells. DeMarco and co-workers [118] published a report that determined the anti-inflammatory properties of *Lactobacillus acidophilus*, *Lactobacillus casei, Lactococcus lactis, Lactobacillus reuteri*, and *Saccharomyces boulardii*. Metabolites produced by the above strains influenced the HT-29 cell line and modulated the level of IL-10, IL-1β, TNF-α, PGE-2, and IL-8. The effect was highly dose-dependent and the strongest modulatory properties were visible for the *Lactococcus lactis* and *Lactobacillus reuteri* medium.

The data confirm that the probiotic strains can suppress the inflammatory state. Protection is demonstrated by signaling path inhibition and the modulation of pro- and anti-inflammatory interleukins. Nevertheless, it is obvious that not only the live bacteria, but the extracted component and even the post-growth media have meaning for the host response. This suggests that the definition of probiotics should be reformulated and expanded by the cell components and bacterial metabolites.

### 4.5. Probiotic and Intestinal Epithelial Barrier Function

The mucus barrier is the first line of host defense against pathogenic bacteria and infections. It is the point of contact between the external environment and the host’s internal milieu. The integrity of the mucus barrier is crucial for the IBD healing process and recovery state [99].

Probiotics are able to strengthen a tight junction within the gut as well as change the T cell subpopulation proportions. Zhang and co-workers published data based on a murine model of DSS-induced colitis and reported that supplementation with a mixture containing *Bifidobacterium, Lactobacillus acidophilus*, and *Enterococcus* (Bifico, Shanghai Sine Pharmaceutical Co., Ltd, Shanghai, China) not only increased the number of Treg and decreased the total number of T cells in the colon and the peripheral blood, but also had a positive influence on the tight junctions [119]. Changes were not observed in the spleen’s Treg population within the study groups. Interestingly, the mice with a previous supplementation of probiotic strains and then (after colitis induction) were treated with Bifico had more severe gut damage than the group that was supplemented with probiotic strains and had induced colitis (without post-induced colitis Bifico treatment). This suggests that severe damage to the intestinal epithelium may not be reversible; probiotic strains can cause damage and can be recognized by the host as a threat. This is in accordance with the case report published in 2015 by Meini and co-workers [120]. An adult patient with severe active UC, hospitalized because of the intensification of the symptoms, was routinely treated with corticosteroids, mesalazine, and antibiotics. After an improvement in their general state, the patient was supplemented with *Lactobacillus rhamnosus* GG. After 13 days of oral probiotic treatment, the patient’s condition had worsened and the patient indicated symptoms of bacteremia. In the peripheral blood, *L. ramnosus* as well as *Candida albicans* were identified. These data suggest that probiotic treatment should be considered individually for each case of colitis.

The data show that the VSL#3 mixture also positively affects the barrier integrity and production of its components. According to Cabarello-Franco and co-workers, the oral supplementation of VSL#3 in the rat model had an influence on the mucus structure and stimulated the expression of the *Muc2* gene, but only slightly on *MUC1* and *MUC3*. Surprisingly, the secretion of the non-mucin glycoprotein also increased. This led to an enrichment of the intestinal mucus content by 60% [99] and improved the permeability of the intestinal barrier. Kumar and co-workers proved that the group of *Muc2*^+/+^ mice supplemented with VSL#3 indicated an intense mucus secretion in the crypts’ goblet cells in the colon, compared to *Muc2*^−/−^ mice, where the crypts’ morphology did not alter. In *Muc2*^−/−^ mice treated by VSL#3, the wall thickness was reduced and the MPO level (myeloperoxidase, a marker of tissue damage in the colon) decreased significantly [121]. Nevertheless, the VSL#3 did not affect the severity of colitis and did not induce changes in colon morphology between the VSL#3 treated and the untreated *Muc2*^−/−^ mice group [121].

Palumbo and co-workers [122] published a report that indicated that the beneficial effect of VSL#3 depends on the source of production. Studies compared the properties of two VSL#3 mixtures produced in different countries (Italy and US) on Caco-2 cell lines. The results of the research were discrepant and indicated the beneficial effect of only one of the products. The permeability of the Caco-2 cells monolayer was analyzed by a Transepithelial Electrical Resistance (TEER) measurement, a flux in FITC-dextran particles, and the expression of zoludin-1 (ZO-1) and occludin. In contrast to the Italian product, the US-manufactured VSL#3 showed a beneficial effect on the tight junctions as well as a protective effect on the epithelial barrier damage caused by induced heat-stress. Moreover, the Italian-made product significantly lowered the level of occludin expression in Caco-2, which exacerbated the permeability of the chosen cell line model. This difference in the usefulness of the same product but manufactured in a different place was also confirmed in the animal models, as discussed above. Furthermore, probiotic bacteria can neutralize some harmful environmental substances, which is also beneficial for the intestinal barrier. Zhai and co-workers ran an experiment on a cell line and a mouse model and examined the ability of *Lactobacillus plantarum* CCFM861 to inhibit heavy metal (cadmium) absorption [123]. The authors reported that *Lactobacillus plantarum* CCFM8610 not only binds the cadmium, but also has an anti-oxidative effect, which protects the intestinal barrier.

Similar to an inflammatory state, the impact of the components derived from probiotic bacteria was proven to affect intestinal barrier permeability. Zakostelska and co-workers [124] proved that modulation properties also have a lysate of *L.casei* DN-114 001. The experiment was performed in two groups: mice BALB/c and SCID. Surprisingly, in the DSS-induced colitis model, the protective meaning of probiotic was demonstrated only on the BALB/c mice. According to the authors, this may underline the meaning of adaptive immunity within the probiotic protection mechanism. Additionally, it was demonstrated that the lysate of *L. casei* increased the barrier function by the upregulation of zonula occludens-1 (ZO-1), increased the amount of Treg cells, decreased the level of proinflammatory factors, TNF-alpha, IFN-gamma, and IL-10, and influenced microbiota composition in mice colons. Gao and co-workers [125] described a new surface layer protein HM0539 from *Lactobacillus rhamnosus GG* (LGG), which was potentially useful for intestinal barrier protection. The monolayer of Caco-2 cells was first incubated with HM0539 (12 h), and then 6h of incubation with LPS and TNF-α was run. After that, the levels of ZO-1, occludin, and *MUC-2* were determined. It was shown that the newly described protein had a protective impact on TJ and increased the level of the tight junction as well as promoting mucin secretion. A similar study was reflected in an animal model with DSS-induced colitis. Once again, improvement in the permeability of the intestinal barrier was confirmed. Nevertheless, the direct pathway of this protection needs further investigation. In other studies, Yin and co-workers [126] isolated the Micro Integral Membrane Protein (MIMP) from *Lactobacillus plantarum*. These particles affected the Caco-2 cell line, reducing its permeability. The experiment was repeated on a murine model of DSS-induced colitis. The authors noted that the permeability was significantly reduced and the expression of JAM-1, occludin, and ZO-1 was significantly increased. These findings suggest that individual components of bacterial probiotic strains can be useful in the protection of the epithelial/mucus barrier: the first line of host defense. The effectiveness of probiotics in the treatment of IBD is summed up in Table 2.

## 5. Conclusions

To summarize, IBD is a very complex disease, and the direct causes and pathomechanisms are not fully known. IBD-related states seem to depend on many internal and external factors including genetic, environmental, immunological, and the macrobiotic state of the organism. Further research is needed to fully understand the mechanism. The use of probiotics seems to be a very promising therapeutic strategy. According to the literature, probiotic bacteria can affect all aspects of IBD pathoetiology, and can fulfil a protective function for the patient. It is necessary to know their path of action and all their properties. It should be noted that many of the publications are based on the animal model of the IBD. The models with rats or mice are widely used, nevertheless, they do not fully reflect the human disease. First of all, the occurrence of IBD is a result of the coexistence of many factors (internal and external as additional diseases, diets, genetic background), which cannot be fully reflected in the lab. An additional limitation is the difference in the organization and function of the gastrointestinal tract between different species. This suggests that the usefulness of the probiotic strains have to be tested on many different research models. In recent years, the properties of bacterial components and metabolites have been distinguished, even introducing a new term “postbiotic”. It seems to be clear that the use of particular components can be safer for the patient. Nevertheless, further research is needed. Supplementation with live strains of probiotics has its pros and cons. The live organism seems to act in a multimode; it stimulates the host cell and actively changes the environment (for example, through the neutralization of heavy metals, changing of the microbiota composition). However, in special cases, it can cause bacteremia or some intestinal damage. In that aspect, the use of a postbiotic seems to more reasonable. Nevertheless, the live probiotic seems to be most beneficial when combined and administered at the same time. This indicates the synergy between different stains. Perhaps the future of gut bacteria-based therapy lies in the use of a mixture of probiotic and postbiotic components.

## Figures and Tables

**Table 1:** The Montreal classification for Crohn’s disease (CD) and ulcerative colitis (UC) [9,10,11].

CD Classification		UC Classification	
Age at diagnosis	A1:< 17 years	Severity	S0: remission, no symptoms
	A2: 17–40 years		S1: mild symptoms
	A3: > 40 years		S2: moderate symptoms
			S3: severe symptoms
Location, endoscopic or macroscopic estimation	L1: terminal ileal	Extensity	E1: ulcerative proctitis
	L2: colon		E2: left-sided UC; distal colitis
	L3: ileocolon		E3: extensive UC, pancolitis
	L4: upper GI modifier: proximal disease with distal disease, such as L1 + L4, L2 + L4, L3 + L4)		
Behavior over time	B1: non-stricturing, non-penetrating		
	B2: stricturing		
	B3: penetrating		
	P: perianal disease modifiers, such as B1p, B2p, B3p		

**Table 2:** The effectiveness of probiotics in the treatment of IBD.

Strain	IBD	Research Model and Study Scheme	Results	Ref.
*Bifidobacterium infantis* (BabyLife, Solaray)	CD-like	Rat model (TNBS) 10 days supplementation with 0.205 g of *B. infantis* dissolved in 1.0 mL distilled water prior colitis. Colitis was induced by enema of 1.0 mL 5% (w/v) TNBS which lasted for 7 days.	Reduction in the symptoms, weaker damage to the mucosal architecture, protective function for mucus goblet cells and the epithelial cell layer;	[75]
Summary: a beneficial effect was observed				
*Bifidobacterium bifidum* PRL2010	CD-like	Murine model (TNBS) 7 days oral supplementation of 10^9^ of *B. bifidum* PRL 2010. Colitis (TNBS 2.5 mg/mice) was induced in the 5^th^ day of probiotic strain feeding.	Reduction in the edema, reduction in the macroscopic damage and histological scores, reduction in weight-loss, anti-inflammatory effect;	[76]
Summary: a beneficial effect was observed				
*Bifidobacterium bifidum* 231	CD-like	Rat model (TNBS) Colitis was induced by 31 mg/kg of TNBS. 14 days supplementation of 1.4 × 10^11^ CFU/rat/day *B. bifidum* (in saline) after colitis induction	Reduction in the edema, reduction in the macroscopic damage and histological scores, reduction in weight-loss, anti-inflammatory effect;	[77]
Summary: a beneficial effect was observed				
*Lactobacillus plantarum* species 299	CD-like	Rat model (TBSN) Colitis was induced by 30 mg (0.6 mL of 5% aqueous solution) of TNBS. 7 days supplementation of 10^9^ colony forming units (CFU) of *Lactobacillus plantarum* (in oat fiber) after colitis induction.	No beneficial effects on the rat’s gut permeability, weight changes, colon microscopic scores, and the level of blood albumins;	[78]
Summary: Lack of positive effect				
*Bifidobacterium animalis* subsp. *lactis* BB12	UC-like	Murine model (DSS) 7 days supplementation (twice a day) with 1.2 × 10^10^ CFU *Bifidobacterium animalis* subsp. *lactis* BB12 by oral gavage prior to colitis. Colitis was induced by 3% DSS added to drinking water for 6 days.	Protection against a reduction in colon length, better picture of the colon histology, reduction in apoptosis in the epithelial layer, decrease in the level of TNF-α;	[79]
Summary: a beneficial effect was observed				
*Bifidobacterium longum* subsp. *infantis* BB-02	UC-like	Murine model (DSS) 10 days supplementation (by oral gavage) once a day with 0.1 mL of a suspension containing 9.0 log_10_ CFU/mL in phosphate-buffered saline prior to colitis induction and continued during its development for next 7 days. Colitis was induced by DSS 3.5% (w/v) in drinking water ad libitum for 7 days.	Reduction in the clinical symptoms of the disease, protection of the colonic structure, reduction in edema;	[80]
Summary: a beneficial effect was observed				
*Bifidobacterium lactis* from fermented milk (Activia; Danone)	UC-like	Murine model (T-bet^−/−^Rag2^−/−^) 100 mg of diary product was orally instilled daily, and additional 100 mg per mouse in its cage was provided for consumption.	Reduction in severity of colitis and inflammation in an early stage, decrease in the level of *Enterobacteriaceae* (colitogenic)	[81]
Summary: a beneficial effect was observed				
*Lactobacillus delbrueckii*	UC-like	Murine model (DSS) Colitis was induced by the addition of 3% (w/v) DSS in the drinking water for 7 days. Administration of probiotic (5 × 10^9^ bacteria/mouse/day started 1 day) before colitis induction and lasted until sacrifice.	Modulation of the NF-kB pathway, reduction in the inflammatory state;	[82]
Summary: a beneficial effect was observed				
VSL#3 (*L*. *paracasei*, *L*. *plantarum*, *L*. *acidophilus*, *L*. *delbrueckii* subspecies *bulgaricus*, *B*. *longum*, *B*. *breve*, and *B. infantis, Streptococcus thermophilus*)	UC-like	Murine model (DSS) 8 days supplementation (by oral gavage) with 0.1 mL of a suspension containing 5 × 10^10^ probiotic CFU/kg of body weight dissolved in saline solution after colitis induction. Colitis was induced by 5% DSS (w/v) in drinking water for 8 days.	Discrepancy in results (two different batches of VSL#3, contrary data); VSL#3 batch A: reduction in macroscopic scores, intestinal permeability, -reduction in expression of TNFα, IL-1β, IL-6 mRNAs, increase in the expression of TGFβ, IL-10, occludin, zonula occludens-1 (ZO-1) mRNAs, shift of colonic macrophages from a M1 to M2 phenotype- lack of effect in VSL#3 batch B	[84]
	CD-like	Murine model (TBNS) Eight days supplementation (by oral gavage) with 0.1 mL of a suspension containing 5 × 10^10^ probiotic CFU/kg of body weight dissolved in saline solution after colitis induction. Colitis was induced by 1 mg of TNBS fasted for 12 h.		[84]
Summary: positive effect only one batch of the same product				
*Bifidobacterium animalis* spp. *lactis* Bl 5764	CD-like	Murine model (TNBS) 5 days supplementation (by oral gavage) of a 5 × 10^8^ CFU/day/mice prior to colitis induction and continued during its development for next 2 days. Colitis was induced by TNBS (110 mg/kg, dissolved in 0.9% NaCl/ethanol (50/50 v/v)).	Lower body weight-loss, better macroscopic indicators of inflammation (Wallace scores), histopathological analysis (Ameho score), and level of lipocalin-2, promotion in the bone marrow-derived dendritic cell maturation and IL-17A secretion	[85]
*Lactobacillus reuteri* Lr 5454	CD-like	Murine model (TNBS) 5 days supplementation (by oral gavage) of a 5 × 10^8^ CFU/day/mice prior to colitis induction and continued during its development for next 2 days. Colitis was induced by TNBS (110 mg/kg, dissolved in 0.9% NaCl/ethanol (50/50 v/v)).	Lower body weight-loss, better macroscopic indicators of inflammation (Wallace scores), histopathological analysis (Ameho score), and level of lipocalin-2; greater involvement in the development of tolerogenic DC, induce Tregs population and expression of Reg3b in a NOD2-independent manner;	[85]
Summary: a beneficial effect was observed				
*Saccharomyces boulardii* (Floratil^®^)	CD	Human model Patients with CD in remission (based on Crohn’s disease activity index) were supplemented with *S. boulardii* about 4 × 10^8^ cells every 8 h as an oral capsule formulation during 3 months.	Help in maintaining remission and bowel sealing;	[86]
Summary: a beneficial effect was observed				
*Bifidobacterium breve* strain Yakult, *Bifidobacterium bifidum* strain Yakult, *Lactobacillus acidophilus* strain Yakult (Yakult Co. Ltd. Japan)	UC	Human model Patients with mild to moderate active UC were supplemented with 100 mL/day (10 billion cells) of bifidobacterial-fermented milk for 3 months.	Promising usefulness in sustaining the remission phase, improvement in clinical activity index score and histological scores;	[87]
Summary: a beneficial effect was observed				
*Escherichia coli* Nissle1917 (Mutaflor 100 mg; Ardeypharm GmbH, Herdecke, Germany)	UC	Human model Randomized, double blind, double dummy trial, patients with UC remission were supplemented with 2.5–25 × 10^9^ viable bacteria for 12 months.	Promising behavior in sustaining the remission phase, prevention from inflammatory state;	[88]
Summary: the beneficial effect was demonstrated				
*Lactobacillus fermentum, Lactobacillus delbruekii*. (Lacteol Fort; Rameda, Egypt)	UC	Human model Patients with mild to moderate UC assessed by Mayo score were supplemented with 10 billion CFU of probiotic cells (powder to dissolve in 50 mL fresh water) for 8 weeks.	Decrease in the IL-6 and TNF-alpha levels and lowering regulation of NF-kB;	[89]
Summary: a beneficial effect was observed				
*Bifidobacterium infantis* 35624	UC	Human model Randomized, double-blind placebo-controlled studies, patients with mild to moderate active UC (based on a clinical activity index) were supplemented with 1 × 10^10^ CFU viable probiotic cells for 8 weeks.	Reduction in the levels CRP and TNF-α in both gastrointestinal and non-gastrointestinal inflammatory disorders, but did not particularly affect UC disease;	[90]
Summary: Lack of effect for UC group, positive effect for gastrointestinal, and non-gastrointestinal inflammatory disorders group				
*Bifidobacterium breve* strain Yakult	UC	Human model Patients with UC (active and inactive) were supplemented with 1 g of the freeze-dried powder containing probiotic (10^9^ CFU/g) for 1 year.	Better endoscopic scores, decrease of MPO level, modulation of luminal environmental factors such as intestinal microflora and pH	[91]
Summary: a beneficial effect was observed				
*Bifidobacterium breve* strain Yakult*, Lactobacillus acidophilus* strain Yakult (Yakult fermented milk (Mil–Mil))	UC	Human model Multileft, randomized, placebo-controlled, double-blind parallel-group study; patients with UC in remission were supplemented with 100 mL/day (10 billion cells of *Bifidobacterium* and 1 billion of cells of Lactobacillus) of fermented milk for 12 months.	No beneficial effect	[92]
Summary: no beneficial effect was observed, the study was discontinued				
*Lactobacillus acidophilus* strain LA-5 and *Bifidobacterium animalis* subsp. *lactis* BB12 (Probio-Tec AB25)	UC	Human model Randomized, double blind, placebo-controlled study; patients with UC in remission were supplemented with 1.5 × 10^11^ CFU daily (2 capsules 3 times daily) for 52 weeks.	Maintaining remission in colitis	[93]
Summary: a beneficial effect was observed				
*Bifidobacterium longum 536* (Morinaga Milk Industry Co. Ltd, Tokyo, Japan)	UC	Human model A randomized, double-blinded, placebo-controlled multileft trial study; patients with mild to moderate UC (based on disease activity index) were supplemented with 2–3 × 10^11^ freeze-dried viable probiotic capsule 3 times daily for 8 weeks.	Decrease in the disease activity index and downscale the rectal bleeding, clinical remission;	[94]
Summary: a beneficial effect was observed				
*Lactobacillus salivarius, Lactobacillus acidophilus, Bifidobacterium bifidum* strain BGN4; Acronelle^®^, Bromatech SRL, Milan, Italy)	UC	Human model Patients with moderate to severe UC (based on disease activity index) were supplemented with probiotic blend for 24 months.	Reduction in recovery time, weaker activity of the disease, better endoscopic picture;	[95]
Summary: a beneficial effect was observed				
VSL#3	UC	Murine model UC associated carcinogenesis model was based on a single injection of 12.5 mg/kg body weight azoxymethane intraperitoneally, 1 week later 2.5% DSS was added to drinking water for 5 days, followed by 10 weeks and 2 days of regular drinking water. Probiotic mixture (1.5 × 10^9^ CFU/mice) was supplemented alone or together with mesalazine.	Decrease in the level of TNF-α and IL-6, reduction of number of pathogenic microbiota, increase in the population of *Bifidobacterium* and other non-pathogenic species in the intestinal mucosa;	[96]
Summary: preventive effect for UC associated carcinogenesis				
VSL#3	UC	Human model Patients with mild to moderate, active UC (based on Activity Index) were supplemented with the probiotic mixture twice daily for 12 weeks.	Improvement in rectal bleeding and stool frequency, mucosal appearance and overall physician’s evaluation;	[97]
Summary: a beneficial effect was observed				
VSL#3	UC	Human model A multileft, double-blind, randomized, placebo-controlled, parallel study, patients with mild to moderately active UC (based on Activity Index) were supplemented with the probiotic mixture for 8 weeks in addition to standard therapy.	Reduction in UCDAI scores and frequency of rectal bleeding; - no differences in parameters such as the physician’s rate of disease activity, or endoscopic scores;	[98]
Summary: a beneficial effect was observed				
VSL#3	UC	Human model, children population Patients with mild to moderately active UC (based on activity index) were supplemented with the probiotic twice daily for 8 weeks with a dose of probiotic based on their age (from one-half sachet to two and one-half).	Remission in colitis, improvement in microbiota composition, decrease in level of IFN-γ, TNF-α, CRP, ESR;	[100]
Summary: a beneficial effect was observed				
*Faecalibacterium prausnitzii*	CD n.a	Murine model (TNBS) A double-blind controlled trial study, 5 days before colitis induction mice were supplemented with 10^9^–10^10^ CFU bacterial suspension or bacterial medium. Colitis was induced by TNBS (100 mg/kg body weight), which was administrated intrarectally. The observation took 20 days.Cell line CaCo-2	Anti-inflammatory effect, blocking of NF-kB pathway and IL-8 production, anti-inflammatory effect;	[103]
Summary: a beneficial effect was observed				
*Bifidobacterium lactis*	Cancer model	Murine model 6 days of supplementation with probiotic (in different dose) prior to colitis. An acute colitis was induced by 3.5% DSS in drinking water for 7 days. Colitis associated cancer was induced by azoxymethane (10 mg/kg) prior to 5 days DSS challenge. Cell line model (HT-29) Cell line HT-29 was incubated with the different concentration of Bifidobacterium lactis.	Inhibition of NF-kB and NF-kB-regulated genes in epithelial cells and prevention meaning for the acute colitis and cancer model, reduction in number and size of the tumors;	[108]
Summary: a beneficial effect was observed				
*Lactobacillus acidophilus Lactobacillus fermentum*	Cancer model	Murine cancer model 12 weeks supplementation with probiotic (0.5 × 10^10^ CFU of each strain)among Apc^Min/+^ mice. Cell line model (CaCo-2) Bacteria (alone and as a mixture) prepared in simulated artificial intestinal juice were incubated with Caco-2 (up to 72 h).	Reduction in cancer cells proliferation, increase in apoptosis level, protection of normal colon cell growth from toxic treatment;	[110]
Summary: a beneficial effect was observed				
VSL#3	Cancer model	Murine cancer model (IL10^−/−^^)^ Supplementation with 1.2 billion bacteria per mouse/day) or conjugated linoleic acid. Cancer was induced by azoxymethane, DSS (the first step) and a single dose of 5 × 10^7^ CFU *Helicobacter typhlonius* by oral gavage (the second step cancer induction). Observation took 68 days.	Shorter recovery time, lower disease severity in an active phase of cancer; VSL#3 treatment- higher mRNA expression of TNF-α, increase in the angiostatin level and Tcells subpopulations; CLA treatment- decreased the level of COX-2;	[109]
Summary: a beneficial effect was observed				
VSL#3	Cancer model	Murine cancer model/IL-10^−/−^ Mice with IL-10 deficient were supplemented by VSL# from the day of cancer induction for 17 weeks (once a day without the weekends). Cell line model (CaCo-2) Bacteria (alone and as a mixture) prepared in simulated artificial intestinal juice were incubated with Caco-2 (up to 72 h).	No protection against inflammatory processes and tumor development, increase in the tumor penetrance, worsening histologic dysplasia scores, extension of Clostridium population;	[112]
Summary: lack of beneficial effect, worsening of the neoplastic processes				
VSL#3	Cancer model	Rat cancer model (TNBS) Supplementation with VSL#3 1 week before the colorectal cancer induction by TNBS. Then rats were supplemented with probiotic in drinking water until end of experiment (17 weeks).	Delay in the carcinoma growing processes, improvement in the histological picture of the colon, increase in the level of angiostatin vitamin D receptor (VDR);	[111]
Summary: a beneficial effect was observed				
*Propionibacterium freudenreichii*, Isolated: SlpB, SlpE, two proteins with SLH domains, HsdM3	n.a	Proteomic and transcriptomic study	Potential connection with IL-10 increase and anti-inflammatory value	[116]
Summary: anti-inflammatory effect as a results of combination cytoplasmatic and surface protein				
*Lactobacillus rhamnosus GG,* isolated antigens: surface layer protein, genomic DNA and unmethylated cytosine-phosphate-guanine-containing oligodeoxynucleotides, alone or in combination.	n.a	Cell line RAW 264.7 Cell line was incubated with bacterial cells, components, or their combination (2 h), then was challenged with lipopolysaccharide (0.5 h).	Suppression in the inflammatory paths, inhibition of TLR, MAPK and NF-κB signaling pathways	[117]
Summary: anti-inflammatory properties were observed				
bacteria’s cells-free growth medium, *Lactobacillus acidophilus, Lactobacillus casei, Lactococcus lactis, Lactobacillus reuteri*, and *Saccharomyces boulardii*	n.a	Cell line HT-29 After LPS challenge (4 h), cell line was incubated with cell free bacterial medium (18 h.)	Anti-inflammatory properties, modulation of the level of IL-10, IL-1β, TNF-α, PGE-2, IL-8;	[118]
Summary: anti-inflammatory properties were observed				
*Bifidobacterium, Lactobacillus acidophilus*, *Enterococcus* (Bifico, Shanghai Sine Pharmaceutical)	CD-like	Murine model (IL-10-deficient, DSS) Colitis was induced by 4% DSS in drinking water by 14 days. Mice were supplemented with 3 × 10^7^ CFU probiotic in all study groups (with colitis or not) for 14 days.	Increase in the number of Treg, decrease in the total number of T cells in the colon and the peripheral blood, positive influence on the tight junctions;	[119]
Summary: a beneficial effect was observed				
*Lactobacillus rhamnosus GG*.	UC	Case report A female patient affected by UC with severe active pancolonic involvement.	Symptoms of bacteremia;	[120]
Summary: probiotic strain as a cause of bacteremia				
VSL#3 (Seaford Pharmaceuticals)	n.a	Rat model 7 days intragastrical supplementation with 3 × 10^9^ bacteria.	Influence on the mucus structure, stimulation of expression of the Muc2 gene, increase in secretion of the non-mucin glycoprotein, improvement in the permeability of the intestinal barrier;	[99]
Summary: a beneficial effect on secretion and mucus within epithelial barrier				
VSL#3	UC-like	Murine model (*Muc2*^−/−^ and *Muc2*^+/+^*; DSS)* 15 days of supplementation with VSL#3. Colitis among Muc2^−/−^ group was induced by 1% DSS added to drinking water for 3 days.	Induction of mucus secretion in the crypts’ goblet cells in the colon, reduction in the wall thickness and MPO level - lack of protection from colitis severity	[121]
Summary: a beneficial effect on mucus secretion and oblet cells, lack of usage in colitis severity protection				
VSL#3	n.a	Cell line Caco-2 Cell line monolayer incubated with 10^8^ CFU/mL (4 h).	Discrepancy in results (two different manufacturers of VSL#3, contrary data) - Italy-made product: increased the permeability of the barrier, and decreased the ZO-1/occludin expression - US-made product: increased the occludin level, pretreatment with VSL#3 prevented the heat-induced epithelial barrier integrity loss	[122]
Summary: different usefulness of the same product but produced by two different manufacturers				
*Lactobacillus casei* DN-114 001, lysate	CD-like	Murine model (DSS) Mice were supplemented with 6 x 10^8^ CFU of heat-killed bacteria, by gavage. The administration of bacteria was repeated every 7 days (4 doses together) prior to colitis. Colitis was induced 7 days later by 3% DSS dissolved in tap water for 7 days.	Protective behavior of probiotic only on the BALB/c mice, increase in the barrier function by the upregulation of ZO-1, increase in the amount of Treg, decrease in the level of proinflammatory factors, TNF-α, INF-γ, IL-10, influence on microbiota composition;	[124]
Summary: a beneficial effect was observed				
*Lactobacillus rhamnosus GG*, surface layer protein HM0539	n.a	Cell line CaCo-2 Caco-2 cell line was incubated with HM0539 (50 ng/mL) for 12 h, then cell line was stimulated using TNF-a, (10 ng/mL) or lipopolysaccharide (1 mg/mL) for 6 h.	Increase in the level of the tight junction, increase in mucin secretion;	[125]
Summary: a beneficial effect was observed				
Lactobacillus plantarum, isolated MIMP	n.a	Murine model (DSS) Mice were supplemented with MIMP (0.1 μg/20 g) for 7 days prior colitis. Colitis was induced by DSS administered in drinking water for 7 days. Cell line Caco-2 Cell line was incubated with MIMP in different concentrations for 48 h.	Reduction in the permeability, increase in expression of JAM-1, occludin, ZO-1;	[126]
Summary: a beneficial effect was observed				
